# History and evolution of blood pressure measurement

**DOI:** 10.1186/s40885-024-00268-7

**Published:** 2024-04-01

**Authors:** Su A Noh, Hwang-Soo Kim, Si-Hyuck Kang, Chang-Hwan Yoon, Tae-Jin Youn, In-Ho Chae

**Affiliations:** 1 Cardiovascular Center, Department of Internal Medicine, Seoul National University Bundang Hospital, 82, Gumi-Ro 173 Beon-Gil, Bundang-Gu, Seongnam-Si, Gyeonggi-Do 13620 South Korea; 2 Department of Internal Medicine, Seoul National University, Seoul, South Korea

**Keywords:** Blood pressure, Auscultatory, Oscillometry, Photoplethysmography, Novel technology

## Abstract

Hypertension is the leading cause of morbidity and mortality worldwide. Hypertension mostly accompanies no symptoms, and therefore blood pressure (BP) measurement is the only way for early recognition and timely treatment. Methods for BP measurement have a long history of development and improvement. Invasive method via arterial cannulation was first proven possible in the 1800’s. Subsequent scientific progress led to the development of the auscultatory method, also known as Korotkoff’ sound, and the oscillometric method, which enabled clinically available BP measurement. However, hypertension management status is still poor. Globally, less than half of adults are aware of their hypertension diagnosis, and only one-third of them being treated are under control. Novel methods are actively investigated thanks to technological advances such as sensors and machine learning in addition to the clinical needs for easier and more convenient BP measurement. Each method adopts different technologies with its own specific advantages and disadvantages. Promises of novel methods include comprehensive information on out-of-office BP capturing dynamic short-term and long-term fluctuations. However, there are still pitfalls such as the need for regular calibration since most novel methods capture relative BP changes rather than an absolute value. In addition, there is growing concern on their accuracy and precision as conventional validation protocols are inappropriate for cuffless continuous methods. In this article, we provide a comprehensive overview of the past and present of BP measurement methods. Novel and emerging technologies are also introduced with respect to their potential applications and limitations.

**Graphical Abstract:**

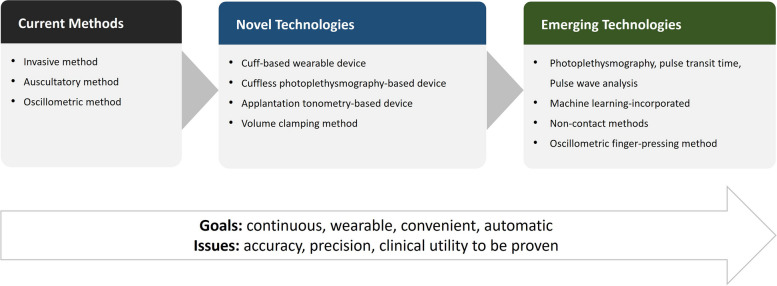

## Introduction

High blood pressure (BP) is the one of main causes of mortality and morbidity across the world [1, 2]. The World Health Organization estimates that approximately 40% of adults have hypertension [3, 4] and that 9.4 million deaths each year are attributable to elevated BP [5]. The prevalence is reported to be 28% among adults aged 20 in Korea [6] with large variations across the world. The number of people with hypertension doubled from 1990 to 2019 globally [7].

BP measurement obtained out of office is gaining importance. BP changes every moment according to the time of a day and the season of a year, and depending on various factors such as temperature, emotion, and physical activity [8]. Single time office BP measurement does not reflect the lifetime track of a person’s BP. White-coat hypertension occurs in 15 to 30% of subjects with an elevated office BP [9], and masked hypertension is estimated to occur in 10 to 30% of adults [10]. Both conditions are associated with a high risk mortality comparable to sustained hypertension [11]. Contemporary clinical guidelines strongly recommend that the diagnosis of hypertension be confirmed by out-of-office BP measurements at least before starting treatment [12–14].

The BP measurement method has a long history in clinical medicine. BP used to be measured in the office using invasive or auscultatory methods. The advent of the oscillometric method has enabled automated and out-of-office BP monitoring. Now consumers can purchase home BP devices at a reasonable price and take BP measurements at their convenience within minutes. Despite such innovations, hypertension management is still far from perfect. Novel methods for more convenient BP measurements are being pursued thanks to advances in digital technologies. Key features include cuffless measurement, wearable devices instead of a bulky machine, and ambient continuous monitoring rather than single time snapshot measurement. Many cuffless devices are present on the market [15]. Each one has a different technology and purpose and a specific pitfall as well.

Novel cuffless devices hold the potential to transform hypertension management. Convenient measurement embedded in wearable devices and smartphones might help find unrecognized hypertension and better identify white-coat and masked hypertension. Continuous monitoring could guide a better lifestyle for hypertensive patients. Detailed information on ambulatory BP and circadian variations would motivate patients with hypertension to be more aware of their treatment status. However, novel technology still has limitations such as the need for calibration at regular intervals with a cuff-based device. Accuracy has been the subject of debate [16]. Lack or paucity of validation studies poses a barrier to widespread use in clinical practice [17, 18].

This review article has three primary objectives: (1) to provide a comprehensive overview of the history of BP measurement technologies and contemporary validation protocols, (2) to examine the evidence for novel technologies currently available in the market, and (3) to introduce emerging technologies with potential applications in the future. Science around BP measurement has a long history. Physicians and scientists have contributed to the development and validation of new technologies and devices. BP is now considered a single reliable treatment target and surrogate marker supported by sound evidence from observational studies and randomized controlled trials. For clinicians who desire to adopt a novel technology into their practice, it is crucial to understand its specific advantages and disadvantages. Scientists who are eager to develop an innovative method also need to appreciate the history of BP measurement and the importance of validation protocols.

## Current methods of BP measurement

**Fig. 1:**
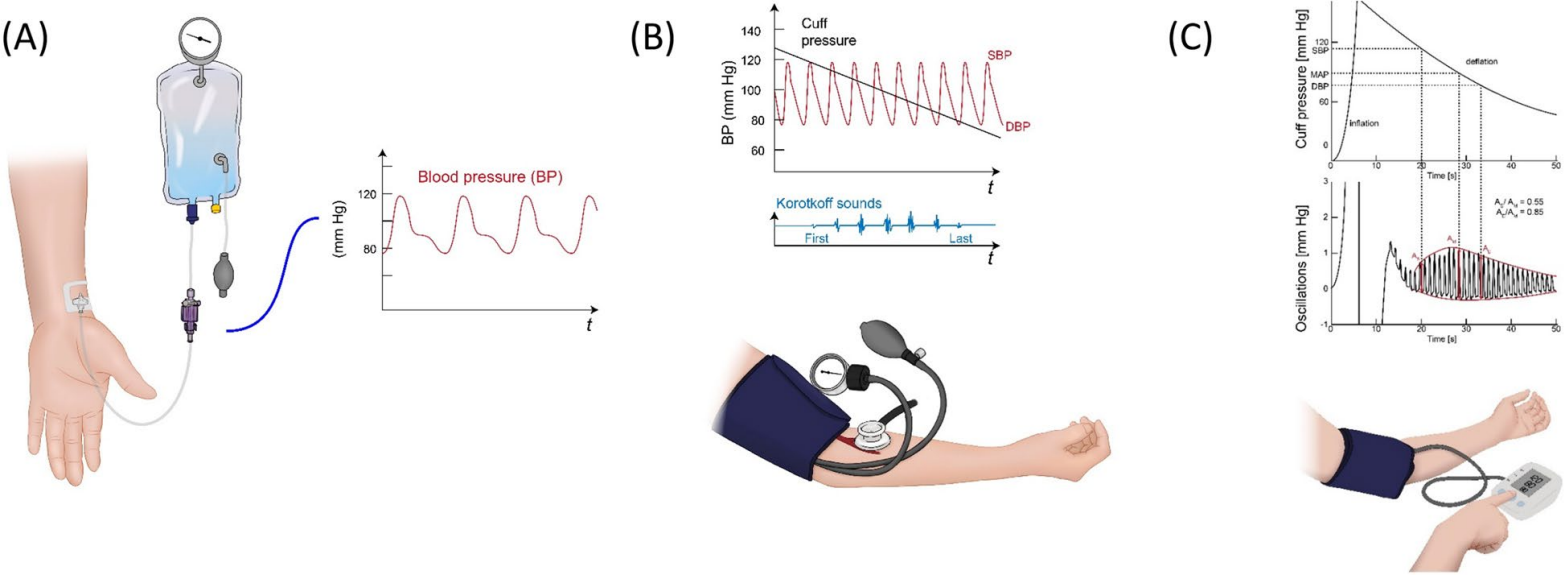
Conventional BP measurement methods. (**A**) Invasive arterial BP monitor, (**B**) Auscultatory method, and (**C**) Oscillometric methods. SBP indicates systolic blood pressure; MBP, mean blood pressure; DBP, diastolic blood pressure

In this section, we will briefly describe BP measurement methods used currently (Fig. 1). History of BP measurement dates back to approximately 300 years ago. Understanding past and contemporary methods is crucial for a critical analysis of the advantages and disadvantages of emerging technologies.

### Invasive method

Since Stephen Hales first reported invasive BP measurement in a horse [19], invasive arterial BP (ABP) monitors have been considered the ground truth as it is the only available method to directly measure BP.

ABP monitoring is an invasive procedure and requires trained personnel. Possible complications include hemorrhage, hematoma, thrombi, infection, vascular insufficiency, arterial injury, accidental intra-arterial infection of drugs and nerve injuries [20]. With the advent of non-invasive methods, ABP monitoring is currently used with limited indications such as in patients who undergo surgery or are admitted to the intensive care units (ICUs) [21, 22]. In addition, it should be noted that ABP measured at the radial artery may not be the same as the aortic pressure or ABP measured at other arteries such as the aorta of brachial artery because of pulse pressure amplification due to the conducting vessel compliance and wave reflection phenomena [23].

### Auscultatory method

Nicolai Sergeivich Korotkoff, a Russian surgeon, reported auscultatory BP measurement using a stethoscope and an inflatable cuff in 1905 [24, 25]. A cuff that encircles the upper arm is inflated above systolic pressure to block the brachial artery blood flow. As the cuff gradually deflates, re-establishment of pulsatile blood flow generates the so-called “Korotkoff sound”. It is commonly thought that the Korotkoff sounds arise from turbulent blood flow through a collapsed artery segment. However, the exact mechanism is yet to be known and there are many other theories for the origin of the sounds [26].

Korotkoff’s important contribution has made non-invasive BP measurement accessible in clinics. However, there are downsides to this method as well. First, this Korotkoff sound method tends to underestimate SBP and overestimate DBP compared to direct intra-arterial BP measurement [27, 28]. It has been shown that, blood flow under the cuff does not begin when cuff pressure falls just below SBP leading to a delay in the generation of Korotkoff sounds [26]. Second, the auscultatory method may not be accurate in patients with severe atherosclerosis. The auscultatory gap refers to the phenomenon of disappearance and reappearance of Korotkoff sounds during cuff deflation usually below the systolic pressure that spans for between 10 and 50 mmHg [29]. It may cause underestimation of SBP, and has been shown to be more frequent in patients with severe vascular atherosclerosis and/or arterial stiffness [30]. Third, there may be inter-observer and intra-observer errors [31].

While the mercury sphygmomanometer had long been regarded as the gold standard, medical use of mercury is now banned in many countries due to safety and environmental concerns [32]. Aneroid and hybrid sphygmomanometers are suitable alternatives [33, 34]. Because their accuracy may degrade over time, they need to be calibrated at regular intervals (commonly every 6 to 12 months).

### Oscillometric method

An oscillometric method has been suggested since 1870’s [35]. Posey et al. discovered in 1969 that the cuff pressure at the maximal oscillation is a reasonable estimate of the mean arterial pressure [36]. Geddes et al. subsequently reported that particular points of the oscillogram correspond with systolic and diastolic BP [37]. They suggested systolic and diastolic pressure at a point when the cuff pressure oscillations were about one half and 0.8 of their maximum amplitude, respectively. Dinamap (an acronym for “device for indirect noninvasive mean arterial pressure”) was the first commercially produced automatic oscillometric BP monitor, which was marketed Applied Medical Research in 1976 [38, 39]. The device was made possible by the emergence of Intel’s first microprocessor and the availability of a new miniature pressure transducer. Since the introduction of oscillometric devices, the theory has been refined and technology has improved over time [40].

As with an auscultatory method, this technique also uses a cuff that encircles and can compress limbs [41]. The main difference is its capability of sensing the parameter identification points and identifying BP values by reading the cuff pressure. Contemporary oscillometric devices mostly acquire mean BP from the maximal oscillation, but the specific algorithm to determine systolic and diastolic BP is proprietary to the manufacturers and kept confidential. Oscillometric methods have made BP measurement available both in and out of office as well as ambulatory BP monitoring without the need for trained persons.

### Validation methods for oscillometric devices

There are variations in the specific algorithms between different manufacturers and different products even from the same manufacturer among oscillometric devices. Each algorithm is kept proprietary and not disclosed outside the manufacturer. No requirements exist to report even when the algorithms are modified by the device manufacturer. Obviously, different devices are not interchangeable and the accuracy and precision of each device need to be validated [42].

Unfortunately, there is no validation requirement for marketing a BP device in the United States [43]. A review article by Cohen et al. nicely explains why the Food and Drug Administration (FDA) clearance (not “approval”) process for BP devices does not mandate validation data [44]. Many devices are sold on the market without rigorous validation, or manufacturers have declined to share validation data when queried [45]. There have been efforts from independent scientific societies to validate BP devices in the market and make the information available to consumers [46, 47].

Main validation protocols for oscillometric devices are summarized in Table 1. Validation criteria for oscillometric devices worth attention for future discussion on novel cuffless devices. The protocols commonly require that test device BP be compared with reference BP measurement, typically an auscultatory method, in the same arm sequentially. Auscultation is performed simultaneously by two trained observers blinded to each other’s readings and to the measurements taken with the test device. Between-observer agreement of ≤4 mm Hg is required to obtain an acceptable reference standard measurement. Four reference standard measurements bracketing 3 device measurements taken in alternating fashion are used for analysis. Criteria for subject selection and methods of analysis are protocol specific.

**Table 1:** Major blood pressure device validation protocols

Protocol	Year published	Sample size required (No. of paired readings)	Criteria indicating a valid device
BHS [48]	1990	85 (255)	Device graded from A to D. Grade A is the highest level of accuracy and requires that the percentage of readings with a difference between the device-under-test and the reference sphygmomanometer of ≤5, 10, and 15 mm Hg be 65, 85, and 95%, respectively.
ESH International Protocol [49]	2010	33 (99)	Pass requirements are split into 2 phases and are based on the number of measurements with differences between the device-under-test and reference sphygmomanometer of ≤5, 10, and 15 mm Hg. See protocol for details. This protocol is being phased out, to be replaced by a joint universal AAMI/ESH/ISO validation protocol requiring 85 subjects.
AAMI/ANSI/ISO [50]	2013	85 (255)	• Criterion 1: when analyzed as 255 paired determinations, the mean difference between the device-under-test and reference sphygmomanometer is < 5.0 mm Hg, and the SD of the difference is < 8.0 mm Hg. • Criterion 2: when analyzed as 85 paired determinations, the SD of the difference between the device-under-test and reference sphygmomanometer is < 4.79 to 6.95 mm Hg (the actual threshold varies according to the mean difference observed. See protocol for details).
AAMI/ESH/ISO [51]	2018	85	• A device is considered acceptable if its estimated probability of a tolerable error (≤10 mmHg) is at least 85%. • The mean BP difference (test versus reference) and its SD, criteria 1 and 2 of the ANSI/AAMI/ISO 81060–2, to be applied for systolic and diastolic BP.

*AAMI* indicates Association for the Advancement of Medical Instrumentation, *ANSI* American National Standards Institute, *BHS* British Hypertension Society, *ESH* European Society of Hypertension, and *ISO* International Organization for Standardization

The AAMI/ESH/ISO standard published in 2018 is now considered the universal standard and most widely used [51]. According to the protocol, a device is considered acceptable if its estimated probability of a tolerable error (≤10 mmHg) is at least 85%. At least 85 subjects are required, and minimum numbers of subjects are suggested according to the cuff size, participants’ sex, and special populations. There are also criteria for mean BP difference (test versus reference) and its SD.

## Novel technologies

Novel methods have emerged to make BP measurement more convenient and easier. The innovations are primarily spurred by technological advances such as sensors, wearable devices, and artificial intelligence. Clinical needs are also playing an important role. Growing body of evidence suggests the importance of out-of-office BP measurements to trace BP variability and to identify masked or white-coat hypertension [12, 52]. BP is a continuous dynamic variable. The interest is shifting from a one-time office BP measurement to continuous lifelong BP monitoring with an analogy of snapshot photographs compared to continuous high-definition video recording [8].

### Cuff-based wearable device

**Fig. 2:**
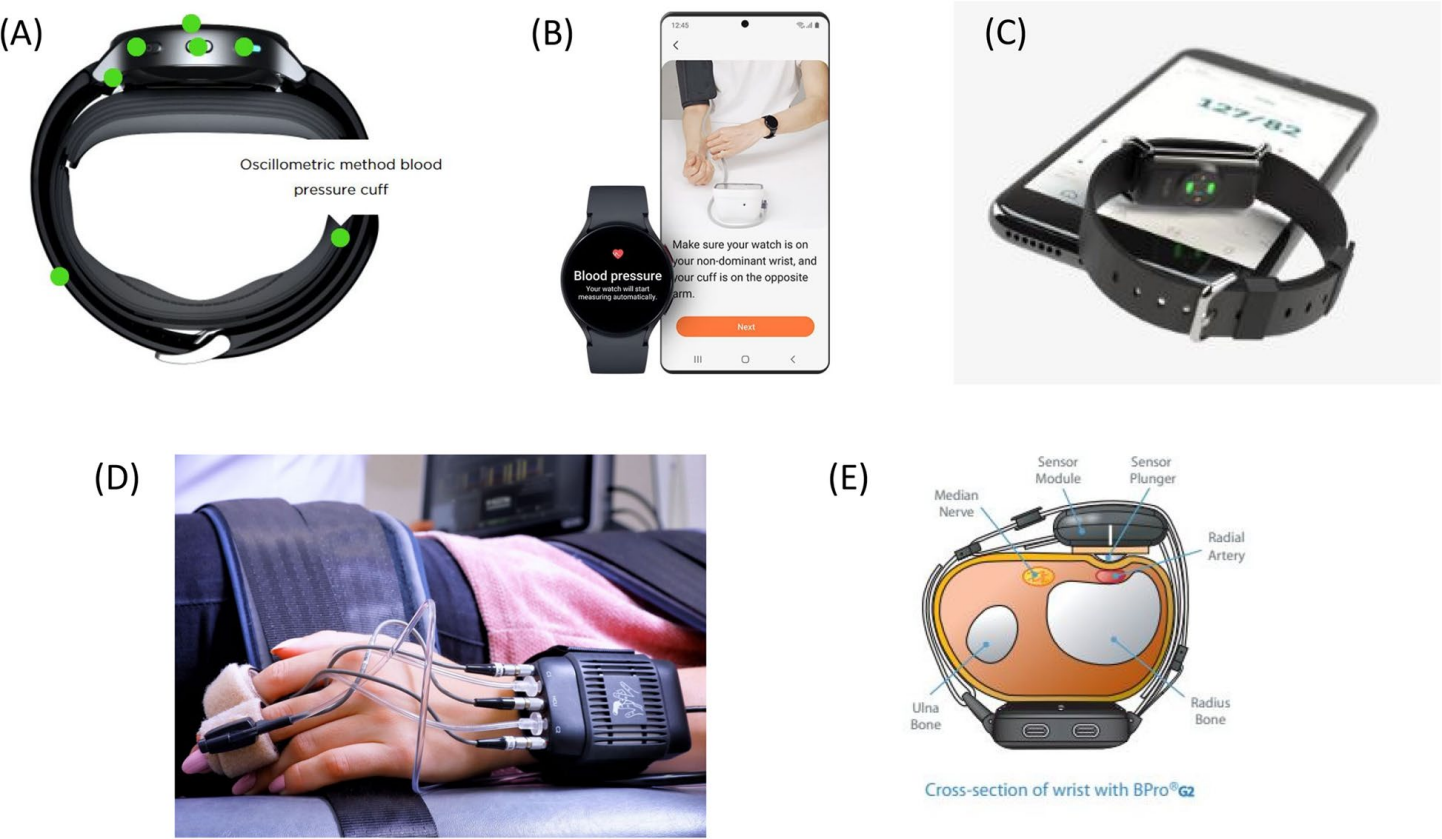
(**A**) Heartguide, a miniaturized version of an oscillometric device (**B**) Galaxy watch, a smartwatch requiring regular calibration with a cuff-based device (**C**) Aktiia, a bracelet using PPG signal (**D**) BPro, a wrist-worn device using modified applanation tonometry (**E**) Volume clamping, a cuff-based ring-type device applying the Peñáz principle

HeartGuide (Omron, Japan) is the first FDA-approved BP watch device (Fig. 2A). The wearable wrist-worn watch-type BP monitoring device works with an inflatable cuff and is basically a miniaturized version of a conventional oscillometric device. BP measurement is activated only when the position sensor of the device judges that the wrist is at the level of the heart. BP readings can be transferred to and displayed on a dedicated app. Users should be careful in positioning their wrist at the level of their heart in order to get an accurate reading. According to previous studies, hydrostatic pressure of 7 mmHg can be caused for every 10 cm difference in height between the heart and the cuff position [53, 54].

Kuwabara et al. validated the accuracy of the device fulfilling both validation criteria 1 and 2 of the ANSI/AAMI/ISO 81060-2:2013 guidelines when compared to BP measurements by a standard mercury sphygmomanometer [55]. Kario et al. also showed that the difference between the watch-type BP monitor and conventional ambulatory BP monitor was acceptable both in and out of the office when the two devices were simultaneously worn on the same non-dominant arm throughout the monitoring period [56]. Based on the study results, HeartGuide was cleared by the FDA for availability as a personal medical device in 2018 [57]. It was also shown that BP falls during (pre) syncope and BP elevations associated with psychological stress can be detected with the device [58, 59]. Another study suggested that the wearable BP monitor might be useful in predicting target organ damage such as left ventricle hypertrophy [60].

Main limitation is poor user experience. The device is bulky and uncomfortable. The strap is thick, and there is a miniaturized cuff beneath it. The gadget is solely for BP monitoring, and does not provide as many functions as contemporary smartwatches do.

### Cuffless photoplethysmography-based devices

Galaxy Watch (Samsung, Seoul) is a consumer electronic device (Fig. 2B). The wrist-worn smartwatch launched an app measuring BP without a cuff in 2019. The mobile app was approved as a software as a medical device by the Ministry of Food and Drug Safety of Korea and received CE mark in Europe in 2020. It has not been cleared by the US FDA as of 2023.

Calibrating with a cuff-based device every 28 days is recommended to ensure watch measurement accuracy. The watch should be worn on the other wrist while calibrating it. Measurement steps are similar to those for an oscillometric device, and the arm and the device should be positioned on the table. And then, users should repeatedly measure BP values three times within 30 minutes to complete the process.

While the exact algorithm of BP estimation has not been disclosed, it appears that BP is estimated from the waveform of reflective photoplethysmography (PPG) [61]. The laser in the near-infrared range around a wavelength of about 980 nm is generated, which is specifically scattered by red blood cells and then collected by an optical sensor in the device. It is unclear if pulse wave velocity (PWV) is used in the application. PPG and PWV will be further discussed later in the article. By using a specific range of light sources, PPG detects periodic blood volume changes in tissues throughout the heart cycle in a noninvasive way [62].

There are concerns regarding the accuracy of the methods. Indeed, changes in PPG that use a specific range of light are correlated with BP changes. However, there are many confounding factors other than BP that determine PPG such as vasoconstriction/vasodilation, absorption and/or reflection by surrounding soft tissue and bones. Environmental factors such as temperature, humidity, and ambient pressure affect vascular smooth muscle tones and thus the vessel diameter. The watch is not fixed in the wrist, so that the location of the light emitter and receiver relative to the vessels varies from time to time. Moreover, a PPG sensor located on the back side of a wrist-worn watch should reflect blood volume changes at the capillary level rather than the radial or ulnar artery [63]. Evidence suggests PPG accuracy may be affected by skin tone, which may lead to racial bias [64, 65].

According to the data the manufacturer submitted to the regulatory body, the app fulfilled the AAMI/ESH/ISO 2018 Universal Standard [66]: the accuracy threshold for a standard sphygmomanometer is a bias and SD of 5 ± 8 mmHg, allowing a tolerable mechanical error of > 10 mmHg in 15% of cases. A real-world study sponsored by the Korean Society of Hypertension enrolled 760 participants who provided 35,797 BP readings and showed the feasibility of smartwatch-based cuffless out-of-office BP monitoring in the real-world setting [67]. The study showed the feasibility of out-of-office monitoring using smartwatch-based cuffless BP measurements. However, this study also raised a question on the stability of BP before and after calibration: the difference of 7-day average BP before and after calibration was 6.8 ± 5.6 mmHg, and it was greater in patients with higher BP. One study in which three wearable devices were tested in each participant sequentially with the reference oscillometric device immediately after calibration showed good correlation and agreement of the Galaxy Watch 3 [68].

Another study collected data from 24-hour ambulatory blood pressure monitoring Samsung Galaxy Watch Active 2 smartwatch simultaneously on each arm in 40 patients. A systematic bias toward a calibration point was demonstrated, which means that systolic BP was overestimated at lower values and underestimated at higher values [69]. An interesting population survey titled “Daily BP measurement with your Galaxy Watch” was done in 1071 respondents in Korea [70]. The study showed that cuffless BP measurement using a smartwatch application was feasible, but that satisfaction on its accuracy was modest in general population users.

Although the Samsung Galaxy app has been cleared as a medical device in Korea and Europe, its role in clinical practice is yet controversial. Recently, the Korean Society of Hypertension released a position paper on smartphone / smartwatch-based cuffless BP measurement [71]. It is acknowledged that there is a considerable discrepancy in high (≥ 160 mmHg) low BP (≤ 60 mmHg) ranges. Cautions should be taken when used in patients with underlying medical conditions such as vascular and heart disease, diabetes mellitus, end-stage renal disease, neurologic disorders such as tremor, pregnancy. The paper also underlines the importance of appropriate and timely calibration.

Aktiia bracelet is a cuffless continuous BP monitoring device that collects PPG from the subject’s wrist using optical sensors (Fig. 2C). The core technology is named optical BP monitoring algorithm, basically a form of pulse wave analysis using PPG obtained from the wrist (further discussed in section 4) [72, 73]. A once-a-month initialization procedure is required for recalibration with the use of a dedicated cuff [74]. A sponsor-initiated trial of 86 participants demonstrated its accuracy satisfying the validation criteria 1 and 2 of AAMI/ANSI/ISO 2013 protocol within a month after calibration [75]. Aktiia received CE Mark as a Class IIa medical device in 2021.

There are multiple publications either sponsored by or directly from the manufacturer. Its accuracy has been tested against the conventional oscillometric technique [76], ambulatory BP monitoring [77], and the invasive ABP from the contralateral radial artery in hemodynamic patients [78]. An experimental study showed the accuracy remained stable across sitting, lying, and standing positions at various device levels relative to the heart, unless the arm is moving [79]. A sponsor-initiated investigational study is underway in the US to compare weekly and monthly BP averages measured manually by traditional home BP monitoring to those measured automatically by the bracelet [80].

Noteworthily, an independent investigator-initiated study revealed that the device did not accurately track night-time BP decline and medication-induced BP changes [81]. Finally, the NEXTGEN-BP is an investigator-driven open-label, multicenter, randomized controlled trial to evaluate the effectiveness and safety of wearable cuffless monitoring in the management of high BP in primary care [82]. The trial will enroll 600 adults with high BP, who will be randomized 1:1 to the intervention of a wearable-based remote care strategy using the Aktiia device or to usual care. The primary outcome is the difference between the two groups in change in clinic systolic BP from baseline to 12 months. The trial results are highly anticipated and are expected to have a remarkable impact on the management of hypertensive patients.

### Applanation tonometry-based device

BPro is a wrist-worn device that captures the radial arterial pulse waveform using modified applanation tonometry (Fig. 2D). The system consists of a monitor located on the dorsal side of the wrist, a sensor module mounted on the radial artery on the ventral side, and a wrist strap designed to firmly hold the sensor system in location. After the sensor plunger is placed on the radial artery, the system is calibrated to the brachial BP. The pulse waveform is transferred from the plunger to the internal pressure sensor, which is then translated into BP values with its proprietary algorithm [83].

The device is for medical use and generates 24-hour ambulatory BP monitor readings. The ambulatory BP monitor has been cleared by the United States FDA and the European CE Mark. The accuracy has been validated according to the 2002 ESH and 2003 ANSI/AAMI protocols [84]. Komori et al. showed fair agreement with the arm monitor in the ambulatory condition, which was stable irrespective of arm positions [85]. Jakes et al. showed acceptable accuracy in pregnancy and gestational hypertensive disorders [86].

### Volume clamping method

Volume clamping is a continuous noninvasive and automatic method applying the Peñáz principle which states that a force exerted by a body can be determined by measuring an opposing force that prevents physical disruption. (Fig. 2E) [15, 87]. A small cuff embedded with a PPG sensor is placed around a finger. Pulsatile BP waveform is acquired by controlling fast inflation and deflation of a finger cuff in combination with PPG. The BP waveform can be measured by clamping the PPG waveform during the cardiac cycle. There are two commercialized systems: Finapres (Ohmeda Monitoring Systems, Englewood, CO, USA) [88] and CNAP devices (CNSystems Medizintechnik, Graz, Austria). A paper by Fortin et al. proposed a ring-type wearable system for BP monitoring, named CNAP2GO, that directly measures BP by using a volume control technique [89].

## Emerging technologies

Despite the introduction of novel devices mentioned above, cuff-based methods are yet considered as the standard. Cuffless devices require individual user calibration and have limitations that they track intra-person BP changes rather than providing absolute BP values. Accuracy has been a subject of debate for all novel methods. It is evident that further technological advancements are needed to make cuffless BP measurement both convenient and accurate. Various investigative approaches will be discussed in this chapter.

PPG is worth further discussion before introducing novel research, because many approaches utilize pulse waveform acquired using PPG or other methods. A light source and a photodetector allow tracking the blood volume change across the course of the light. Different sources of light can be used according to the purpose. For example, even smartphones flash as a light source and the camera as a detector has shown to reliably measure heart rate [90]. Pulse oximeters usually use two light-emitting diodes that emit light at the 660 nm (red) and the 940 nm (near-infrared) wavelengths as a light source [91]. Oxyhemoglobin and hemoglobin have different absorption spectra at these particular wavelengths, and peripheral oxygen saturation can be calculated from the ratio of absorption. Green light (peak wavelength around 520 nm) is more often used in recent wearable devices, such as wristbands and smartwatches. Green light is well absorbed by hemoglobin and the absorption is relatively constant over a wide range of oxygen saturation levels, which makes it less prone to motion artifacts [92]. However, there are concerns that the tissue penetration depth of green light is shallower than that of red or near-infrared light [93]. Green light reaches the epidermal-dermal junction and papillary dermis, whereas red and NIR light can reach dermal, subcutaneous, and deeper layers. Green light penetration is not deep enough to reach large blood vessels, and may reflect blood flow changes only in the capillary levels.

Pulse transit time (PTT) is the most widely studied variable that can be derived from PPG. PTT refers to the time taken for the arterial pulse pressure wave to travel from the aortic valve to a peripheral site. The R wave on the electrocardiogram (ECG) and the peak of the PPG at the periphery are commonly used [94]. It has long been known that PTT is inversely proportional to BP [95]. PTT has attracted much interest as a cuffless BP estimation method mainly owing to its convenience [96]. Such calculations are basically based on assumptions that the artery is a passively thin-walled, purely elastic tube [97]. However, there are many confounding factors. PTT is influenced by heart rate, BP changes and the compliance of the arteries [98]. The vessel wall varies from person to person. The BP-PTT relationship is a complex and non-linear relationship [99]. Different algorithms such as calibrations, complex regression, and sometimes machine learning are used. There are many anatomical variations measuring PTT: the fingertip can is used when pulse oximetry is used, the wrist for watch-type devices and the chest for longer-term monitoring patch devices. Besides PPG, applanation tonometry [100], radar [101], ultrasound [102], and pressure sensors [103] have been used for pulse waveform acquisition. Pulse arrival time (PAT) and PWV are similar concepts often used interchangeably. PAT is calculated as the time from the ECG’s R wave to the starting point of the PPG pulse wave. PWV refers to the velocity of the pulse transit, the distance divided by PTT. Two major vessels such as the carotid and femoral arteries or the brachial and ankle arteries are used, and PWV is generally considered as a surrogate marker for arterial stiffness.

Pulse wave analysis (or pulse waveform analysis, PWA) is a broader term for processing and extracting features from PPG waveforms, which are known to reflect various physiologic factors, including left ventricular stroke volume, aortic compliance, vascular resistance, and wave reflection phenomena [104]. Speaking of PWA, readers who are familiar with central BP may recall an algorithm for estimating the central waveform from peripheral pressure waveforms. Central BP estimation applies the generalized transfer function to directly measured peripheral BP using cuff devices [105]. In the meantime, various calculations and assumptions are applied for BP estimation from pulse waveforms. The derivatives of the quadratic function are readily accessible values. With the advance of computer technology and data analysis tools, machine learning has enabled more sophisticated analysis [106]. A recent paper from Sky Labs Inc. (Seongnam-si, Korea) reported development of deep learning algorithms using invasive arterial BP data from independent 25,779 surgical cases, called PPG2BP-Net, and validation of its low mean error among separate 629 subjects [107]. It appears that Sky Labs is intending to deploy the algorithm into their ring-type PPG device, CART-I BP, which was cleared by the Korean Ministry of Food and Drug Safety in Aug 2023 [108]. However, the PPG data in the vitalDB database is acquired from finger tips using medical-grade transmissive pulse oximetry devices. It needs to be tested by future studies to see how well the algorithm works for a ring-type green-light reflective PPG device.

**Fig. 3:**
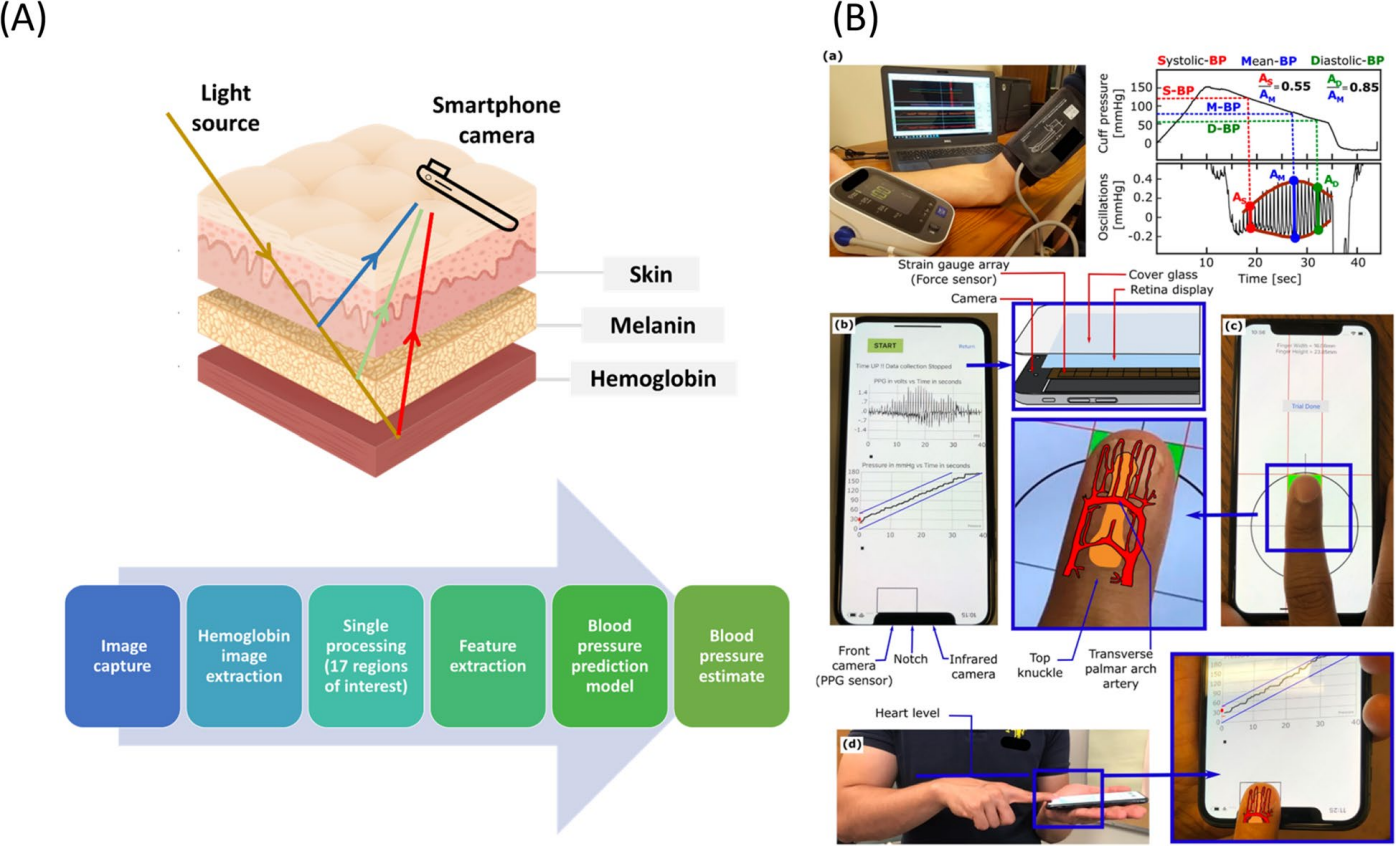
Emerging technologies (**A**) Smartphone-based blood pressure measurement using transdermal optical imaging technology [110]. Licensed … (**B**) An iPhone application for cuff-less and calibration-free blood pressure monitoring via extension of the oscillometric cuff measurement principle [112]. Licensed under CC BY 4.0

Non-contact methods have been developed, most of which capture PPG signals from videos from face or other body parts. Readers who are interested can find more information in a review article [109]. Intuitively, non-contact video methods must be more susceptible to noise and artifacts than contact methods. Luo et al. reported an interesting study that BP can be reliably determined in a contactless manner using a smartphone-based technology called transdermal optical imaging (Fig. 3A). Imperceptible facial blood flow changes are captured with a smartphone camera and advanced machine learning is used to determine BP [110]. Among 1328 enrolled adults, the reported accuracy and precision were acceptable. However, the study subjects were all normotensive and the range of BP was narrow (systolic BP between 98 and 138 mmHg; diastolic BP between 58 and 88 mmHg). The algorithm should be validated in a separate study population with a diverse BP range. D. Yang et al. demonstrate that higher BPs were underpredicted whereas lower BPs tended to be overpredicted [111]. Bias from skin color is another potential concern.

Mukkamala’s oscillometric finger-pressing method is a genius approach that is cuffless and calibration-free using a smartphone (Fig. 3B) [112]. The front camera of a smartphone is used as the PPG sensor, the user serves as the actuator (instead of the cuff) by pressing her/his finger against the camera, and the strain gauge array under the screen (present in the latest iPhone models) is applied as a pressure sensor. First a user presses his/her fingertip against a designated area on the screen and gradually increases the pressure as the app provides visual feedback to guide the amount of finger pressure applied over time. The purpose of this step is to train the user to be able to work as a reliable actuator as the cuff of an oscillometric device does. Next the user’s fingertip is pressed against the front camera in the same way as trained in the previous step. The blood volume changes in the fingertip capillaries are recorded by the camera in an oscillation [113]. The algorithm computes BP values using algorithms similar to the oscillometric method. The device yielded errors comparable to the volume-clamping technique [114]. While the idea of cuffless oscillometric BP measurement using a smartphone is intriguing, the application appears to be less user-friendly and more complex to use than conventional cuff-based oscillometric devices.

Whereas classic cuff-based devices measure absolute BP, many cuffless devices measure BP changes, require periodic calibrations with cuff devices, and/or provide automated BP measurements with sensors not positioned at the level of the heart. Cuffless BP devices have specific accuracy issues, which make the established validation protocols for cuff-based BP devices inadequate for their validation [115]. If a PPG-based device undergoes calibration and follows the conventional protocols, which involve conducting alternative test and reference measurements three times as recommended, the PPG signal should exhibit minimal changes during the validation process leading to stable BP values. Furthermore, many studies introducing new technology typically involve normotensive patients within a narrow age and BP range. The Microsoft Research Aurora Project provides important insight into cuffless BP device validation [116, 117]. A total of 1125 adults were included with a wide age range, both male and female, and multiple hypertensive categories. Multiple cuffless algorithms were compared with simultaneously collected auscultatory or oscillometric references. The result was striking: cuffless BP devices added no value in estimating BP values to baseline models where no actual measurement was used. The “zero baseline” assumed the same value as the initial calibration, and the “static baseline” combined the initial visit calibration values and statistically expected fluctuations according to the time of day. In addition, errors were greater for study populations other than young normotensive patients.

There are increasing needs for validation protocols dedicated for cuffless BP devices. It is needed to define demographically diverse populations and to include dynamic evaluation settings with specific conditions for each device category. Scientific societies are rigorously developing validation protocols for cuffless BP devices [118–120]. The 2023 ESH recommendation proposes six validation tests for these methods: static test, device position test, treatment test, awake/asleep test, exercise test, and recalibration test. The six tests are intended to address all types of cuffless device, thus each type of device is recommended to undergo a different subset of the tests. As discussed above (section 2–4), there are no regulatory requirements for validation studies even for oscillometric devices. Regulatory-cleared devices do not always publish their validation data, and many other devices are sold in the market as a consumer device. Picone et al. revealed that among the top 100 best-selling BP devices on a popular online site, 79% of upper arm and 83% of wrist cuff devices have not been validated [18]. Poor BP data acquired by low performance devices may have negative effects on clinical decision, hypertension management, and patient-physician relationships.

## Conclusion

The evidence around hypertension and BP management has been accumulated as measured by the auscultatory and oscillometric methods. Although oscillometric methods enabled home and ambulatory BP monitoring, the BP measurement process is yet felt uncomfortable. Efforts are being made to develop more convenient novel BP measurement methods. They are mainly cuffless, and mostly provide continuous BP monitoring. In this article, we reviewed the adopted technology of each method and discussed its promises and limitations. Besides HearGuide, a cuff-based wearable device, it has been about 4–5 years since the cuffless PPG-based devices appeared on the market. The number of devices available in the market is increasing rapidly. Novel cuffless approaches have potential to improve hypertension awareness, treatment, and control.

Following the initial excitement, however, there are now growing concerns on their accuracy and precision. There are ongoing efforts to develop internationally accepted validation protocols for cuffless BP devices [118–120]. In addition, inconvenience still exists for novel devices. Because most cuffless devices do not measure absolute BP value and track BP changes compared to the baseline BP, they need calibration at a regular interval with an oscillometric device. Critics may name it “cuff-dependent” rather than “cuffless”.

Clinical value of novel devices for diagnostic or treatment decisions is yet unclear. Patients are increasingly interested in novel BP devices, many of which are sold in the market as a consumer device. Novel devices are strongly recommended to undergo validation studies and publish the data in order to convince physicians and patients. Observational studies assessing the prognostic value of BP measured by a novel device and randomized controlled trials with BP measured with these devices would provide better confidence and demonstrate their clinical utility.

## Data Availability

Not applicable.

## Abbreviations

BP: Blood pressure
ABP: Arterial blood pressure
ICU: Intensive care unit
SBP: Systolic blood pressure
DBP: Diastolic blood pressure
AAMI: Association for the advancement of medical instrumentation
BHS: British hypertension society
ESH: European society of hypertension
ISO: International organization for standardization
ANSI: American national standards institute
FDA: Food and drug administration
PPG: Photoplethysmography
PWV: Pulse wave velocity
PTT: Pulse transit time
ECG: Electrocardiogram
PAT: Pulse arrival time
PWA: Pulse wave analysis or pulse waveform analysis
